# A Workflow for Automated Satellite Image Processing: from Raw VHSR Data to Object-Based Spectral Information for Smallholder Agriculture

**DOI:** 10.3390/rs9101048

**Published:** 2017-10-14

**Authors:** Dimitris Stratoulias, Valentyn Tolpekin, Rolf A. de By, Raul Zurita—Milla, Vasilios Retsios, Wietske Bijker, Mohammad Alfi Hasan, Eric Vermote

**Affiliations:** 1 Faculty of Geo—Information and Earth Observation (ITC), University of Twente, 7514 AE Enschede, The Netherlands; v.a.tolpekin@utwente.nl (V.T.); r.a.deby@utwente.nl (R.A.d.B.); r.zurita—milla@utwente.nl (R.Z.—M.); v.retsios@utwente.nl (V.R.); w.bijker@utwente.nl (W.B.); 2 GeoAnalysis, Budapest, 1134 Hungary; 3 Department of Civil and Environmental Engineering, University of Rhode Island , Kingston, RI 02881, USA; mohammad_hasan@uri.edu; 4 NASA Goddard Space Flight Center, Terrestrial Information Systems Laboratory, Greenbelt, MD 20771, USA; eric.f.vermote@nasa.gov

**Keywords:** automated processing, workflow, real time, very high spatial resolution, surface reflectance, satellite image, smallholder farming, agriculture

## Abstract

Earth Observation has become a progressively important source of information for land use and land cover services over the past decades. At the same time, an increasing number of reconnaissance satellites have been set in orbit with ever increasing spatial, temporal, spectral, and radiometric resolutions. The available bulk of data, fostered by open access policies adopted by several agencies, is setting a new landscape in remote sensing in which timeliness and efficiency are important aspects of data processing. This study presents a fully automated workflow able to process a large collection of very high spatial resolution satellite images to produce actionable information in the application framework of smallholder farming. The workflow applies sequential image processing, extracts meaningful statistical information from agricultural parcels, and stores them in a crop spectrotemporal signature library. An important objective is to follow crop development through the season by analyzing multi—temporal and multi—sensor images. The workflow is based on free and open—source software, namely R, Python, Linux shell scripts, the Geospatial Data Abstraction Library, custom FORTRAN, C++, and the GNU Make utilities. We tested and applied this workflow on a multi—sensor image archive of over 270 VHSR WorldView—2, —3, QuickBird, GeoEye, and RapidEye images acquired over five different study areas where smallholder agriculture prevails.

## 1 Introduction

The world’s population reached 7.3 billion people in mid—2015 [1] and is expected to increase to 9.6 billion by 2050 [2]. Consequently, global demand for food by that year is predicted to double at least that of 2005, not only because of population growth, but also because of a shift to nutrient—richer diets in especially developing nations [3]. The latter scenario calls for technical solutions that help to improve crop yield, provide accurate information to aid in—field management decisions, increase the efficiency of applications of farm inputs, and boost profit margins in the agricultural sector [4]. In some high—income countries, the technology to aid this decision—making may have been established; however, in low—income countries, which are the most populous parts of our planet and which depend strongly on agriculture, information on crops and farm management practices is still primitive.

Remote sensing has been recognized as an important source of information over the last decades in a wide spectrum of Earth Observation applications. The requirements for agriculture—oriented remote sensing systems have long been outlined [5] as the frequency of coverage, high spatial resolution (5 m to 20 m), timeliness, and integration in models. Of these, timeliness is considered the number one requirement as meeting it allows addressing identified problems in real time. Over the past decade, these requirements have been increasingly met by a fleet of reconnaissance satellites with advanced capabilities that allow cost—effective agricultural applications [6].

Moreover, various space agencies and satellite product providers have adopted a free and unrestricted data access policy; for instance, the European Space Agency (ESA) for the Copernicus programme (including the Sentinel—1 and Sentinel—2 satellites) exercises a free, full, and open data policy. Since 2008, the U.S. Geological Survey (USGS) has been providing open access to over five million Landsat images from 1972 onwards. The Japan Aerospace Exploration Agency (JAXA) and the National Aeronautics and Space Administration (NASA) recently made their 30 m Digital Elevation Models (DEM) freely available. The VITO Vision website offers access to PROBA—V and SPOT—Vegetation, and the National Oceanic and Atmospheric Administration (NOAA) is committed to full and open data access. Several partnerships between governments and institutes such as the Brazilian National Institute for Space Research (INPE) Image Catalog and the University Navstar Consortium (UNAVCO) have created catalogues with available free images, and international bodies have been dedicated to the exchange and open—access of ocean— and climate—related data such as the International bodies Global Earth Observation System of Systems (GEOSS), the Committee on Earth Observing Satellites (CEOS), and the World Data System (WDS) of the International Council of Science (ICSU). This policy shift, along with simultaneous developments in image processing software, has fostered easier access to satellite data and lower costs of image deployment. Despite the fact that the image—processing steps are well studied, few of them are fully automated. This can be attributed to their complexity and the required know—how from the user. As we are entering the big data era, the need to establish operational image workflows that produce actionable information in a trusted, robust, and stand—alone fashion is arising.

One application in which Earth Observation can be an important information source is smallholder farming. The fine spatial resolution required to sense smallholder farm fields, the radiometric resolution to discriminate between plant types in this heterogeneous environment, and the temporal resolution required to monitor events and developments (farm practices and crop growth, for instance) during the crop season has become available lately from satellite sensors.

**Figure 1:**
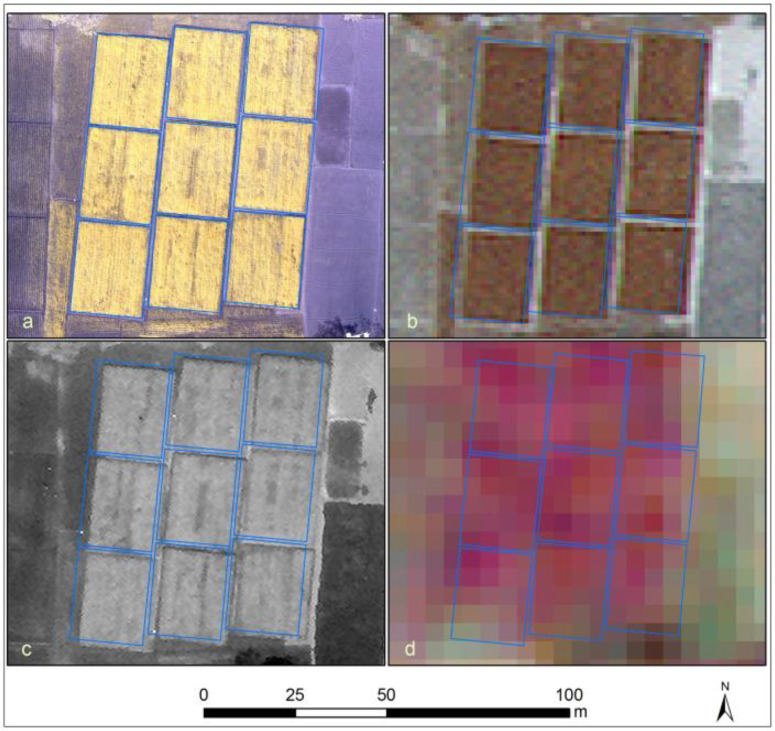
Example of smallholder fields in Barisal, Bangladesh, as depicted by different remote sensors: (**a**) Unmanned Aerial Vehicle (UAV) (Tetracam five—band mini—MCA (multi—camera array) false—colour composite), (**b**) WorldView—2/3 multispectral false—colour composite, (**c**) WorldView—2/3 panchromatic, and (**d**) RapidEye false—colour composite. The overlay parallelograms indicate nine adjacent farm fields. The apparent misalignment between the satellite images at (**b**–**d**) and the polygons are a result of the relatively low horizontal positional accuracy with which the satellite images were delivered.

Earth Observation has fundamentally contributed to large—scale agricultural information systems in high—income countries for some time, especially for tasks in crop scouting for diagnosing soil fertility, the occurrence of insects, diseases, and weed and water problems; monitoring vegetation growth; and estimating potential yield, e.g., [7]. While crop scouting is time— and labor—intensive and thus expensive, Earth Observation presents a viable alternative, even though few studies have reported economic benefit estimates and evidence of on—farm profitability remains fuzzy [8]. The agricultural information landscape in low—income countries is contrastingly different. For one, few farmers have access to important farm inputs, whether products or management recommendations. Consequently, crop production often just covers the basic nutritional needs of the family. Secondly, mixed—cropping and inter—cropping are common practices in smallholder fields, and these practices increase land use intensity. This sketches a landscape in which the established management techniques and data sources of large—scale farming systems are essentially inapplicable. Low—income farming is dominated by small farms that are family—run. Farm size in these areas decreased in the period from 1960 to 2000 [9], and quite possibly the same happened to the average farm field size. This calls for the use of fine spatial resolution images to accrue a sufficient number of pixels per field (Figure 1). While, on the one hand, this will eliminate pixels covering multiple fields and reduce errors in field delineation, increased ground sampling frequency is also known to lead to an increased within—class variability in crop classification. Therefore, spatial resolution requirements differ considerably over different landscapes, even in the framework of a given application [10]. As a consequence, determining the optimal ground sampling distance for an application is a task of identifying the coarsest acceptable pixel size, given the specific landscape.

To monitor crop development throughout the season, a time series of image acquisitions is required. Single satellite platforms cannot trivially satisfy such needs, especially where cloud presence is a limiting factor. For instance, [11] describes the observation requirements for a global agricultural monitoring activity and claims that no current satellite platform with fine—to—moderate spatial resolution can accommodate it. However, a synergistic use of data from multiple agencies and missions may meet the requirements; this leads to the idea of a virtual satellite constellation, which forces significant development in image analytics, especially in the harmonized use of Earth Observation information and the on—time availability of ready—to—use products.

The two requirements of high spatial and high temporal resolution lead to secondary dependencies for our specific application; first, images in a time series must be georegistered with very high accuracy, and, secondly, surface reflectance products acquired at different times and possile, possibly by different platforms, must be carefully spectrally calibrated.

A number of automated image processing methods have been developed in the past. For instance, STORM [12] is a successful example initially developed for 6.5 m RapidEye processing, though challenges surfaced when the workflow was used to tackle 2 m WorldView—2 (WV—2) images. These challenges were encountered especially during geometric correction and were attributed to the finer spatial resolution. While considered successful, the workflow depended on proprietary software (i.e., IDL and ATCOR), the licenses of which are not always available in image processing laboratories, especially when used in low—income countries, where smallholder farming prevails. A similar automatic processing and analysis system for multi—sensor satellite data, named GeoMultiSens, is currently under development [13]. The system emphasizes the analysis of heterogeneous and multi—sensor images on a global scale. A system developed by [14] requires little human interaction to derive vegetative phenological metrics from traffic webcams. Another work, presented in [15], focuses on training and validating satellite data; it, however, requires the user to select reference data to establish an operational system for crop type maps. Last but not least, Clewley et al. [16] built a modular system accessed through Python to conduct Geographical Object—Based Image Analysis (GEOBIA) as an open—source package with functionality similar to existing GEOBIA packages. Other remote sensing domains have also encompassed efforts for automation; for instance, Grippa et al. [17] developed a semi—automated and open—source processing chain for urban object classification.

Next to specialized automated workflows, more general—purpose platforms that offer access and processing capabilities have been released lately. Google Earth Engine is a platform that offers parallel computing, allowing a tremendous speed—up of processing workflows [18]. The DigitalGlobe (DG) company (now part of MDA Corporation) has the GBDX platform for computing with their images, which allows the user to acquire post—processed results instead of purchasing the data, a fact which addresses the concern of cost—effectiveness in obtaining derived image products [19]. ImageQuerying is yet another system for automatic, near—real—time image information extraction from Big Earth data [20]. This system helps the user, through an easy—to—use graphical user interface, to perform semantic content—based image retrieval and to build decision rules with spatial and temporal operators. More generic cloud computing platforms are also available such as the Amazon EC2 [21] and the Microsoft Azure [22].

Despite the fact that many processing systems are available, as presented above, many of them will either not be able to ingest Very High Spatial Resolution (VHSR) data, not be based on free and open—source software, or not meet the requirements of agriculture remote sensing. The Spurring a Transformation for Agriculture through Remote Sensing (STARS) project aimed to investigate the potential of remote sensing in assisting smallholder farming in sub—Saharan Africa and Southeast Asia [23]. To this end, we developed an automated processing chain that ingests VHSR satellite images and derives spectral and textural information for each smallholder field known to the system. This paper aims to showcase the established workflow and demonstrate its potential. We discuss algorithmic development and present examples of products derived by concentrating on three functions of the workflow, namely to:

1. Produce higher processing level products, which can support various scientific purposes. The surface reflectance products aim specifically at vegetation studies.
2. Prepare the data for object—specific statistical information extraction to derive on—demand results and feed a spectral library.
3. Showcase the feasibility of an automated workflow, as a case study of monitoring smallholder agriculture farms from space.

## 2 Materials and Methods

### 2.1 Data

Smallholder agriculture is a remote sensing application that requires very fine spatial resolution data. Popular satellite image types such as Landsat or MODIS are inadequate to cover smallholder farming events with their coarse nominal pixel resolution. To study this dependency, Whitcraft et al. [24] used 3—m resolution images to calibrate a neural network for the sub—pixel classification of 30—m resolution images for small—scale farming in West Shewa, Ethiopia; the results were unsatisfactory. With typical smallholder parcel sizes smaller than 2 ha [25,26], images with much finer spatial resolution than 30 m are required to map within—field vegetation. This would possibly allow the user to discriminate between mono—cropping and mixed cropping, as well as to detect the weed status.

Moreover, agriculture is mostly a seasonal phenomenon and thus requires observations spread over time. The revisit time of single reconnaissance platforms such as Landsat (every 16 days) hold little guarantee for the number of images during the crop season, the more so because most smallholder farming is rainfed and thus takes place in the rainy season, leading to higher chances of cloud presence. Multiple image sources increase the chances to acquire more observations. Hypertemporal imaging, a term coined to indicate a pool of multi—temporal data collected frequently enough to capture the phenomenon dynamics [27], is possible in most cases of optical remote sensing only by making use of a virtual constellation.

Given the above conditions, we conducted a tasked acquisition of high spatial resolution satellite images (Table 1) over two consecutive years (2014 to 2015) and within an active time window, expressed as the start and end year according to the agricultural conditions of each area.

**Table 1:** Technical specifications of Very High Spatial Resolution (VHSR) satellite images ingested by current automated workflow

	Quickbird	GeoEye	WV—2	WV—3	RapidEye
Provider	DigitalGlobe	DigitalGlobe	DigitalGlobe	DigitalGlobe	RapidEye/BlackBridge
Dynamic range	11 bit	11 bit	11 bit	11 bit (14 bit for SWIR)	12 bit
Panchromatic resolution (nominal)	0.65 m	0.46 m	0.46 m	0.31 m	—
Multispectral bands	4	4	8	8 (+8 SWIR)	5
Multispectral resolution (nominal)	2.62 m	1.84 m	1.84 m	1.24 m (3.70 m)	6.50 m
Blue, Green, Red, Near—IR 1	•	•	•	•	•
Cyan (coastal), Yellow, Near—IR 2			•	•	

**Figure 2:**
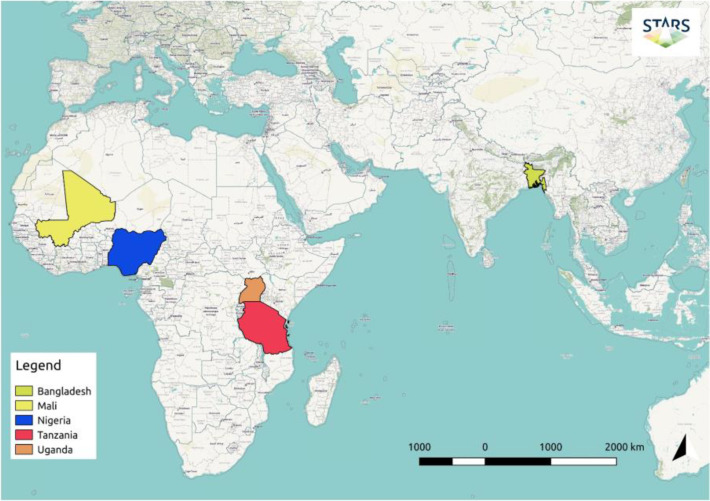
Areas of study in Southeast Asia and sub—Saharan Africa for which VHSR satellite images were acquired and on which the Spurring a Transformation for Agriculture through Remote Sensing (STARS) workflow has been tested. In Mali and Nigeria, over 150 fields of various crops were monitored to examine the ways in which the data can be used to the benefit of the local farming communities. In Tanzania and Uganda, four separate large blocks of croplands were monitored to provide data to the National Food Security office to more accurately forecast crop yields. In Bangladesh, the floodplains of the southern delta were studied to establish irrigation systems that enable farmers to grow a second crop during the dry season.

Use case sites in four countries were targeted in sub—Saharan Africa and one in Southeast Asia (Figure 2). The environmental, agricultural, and landscape conditions varied between them. Two data delivery pipelines were established: one for RapidEye/BlackBridge (now Planet), providing acquisitions from the RapidEye constellation, and another for DG, delivering tasked acquisitions of their optical satellites, namely WV—2, WV—3, GeoEye—1, and QuickBird (Table 1). The latter satellite was in operation until January 2015. The full data bundles were delivered for each sensor, and, for the use case in Bangladesh, the WV—3 SWIR bands were also provided. The project thus resulted in an initial image collection size of 4.5 TB.

### 2.2 The STARS Image Processing Workflow

Our image processing workflow is based on four principles. It

1. is founded on free and open—source software,
2. requires minimal user interaction,
3. supports VHSR satellite image processing, and
4. is tailored to smallholder farming applications.

To adhere to these, the processing is scripted largely with R statistical programming language [28], custom Fortran and C++ utilities, Python, Linux shell scripts, GNU’s Not Unix (GNU) Make, and third party libraries such as GDAL (Geospatial Data Abstraction Library). The system is set up on a server equipped with 8 × 8 GB RAM memory, dual Intel Xeon E5—2640 v3 processors (2.6 GHz), and two 400 GB SSD, and it runs on a Debian Linux operating system. The source code is freely available at the STARS GitHub site (https://github.com/GIP—ITC—TwenteUniversity/stars—image—processing—workflow). The images are archived and processed from a Network Attached Storage (NAS) server with a total storage capacity of 50 TB, which is also the platform of dissemination to end—users. Table 2 lists representative samples of satellite images and their spatial and pixel dimension.

**Figure 3:**
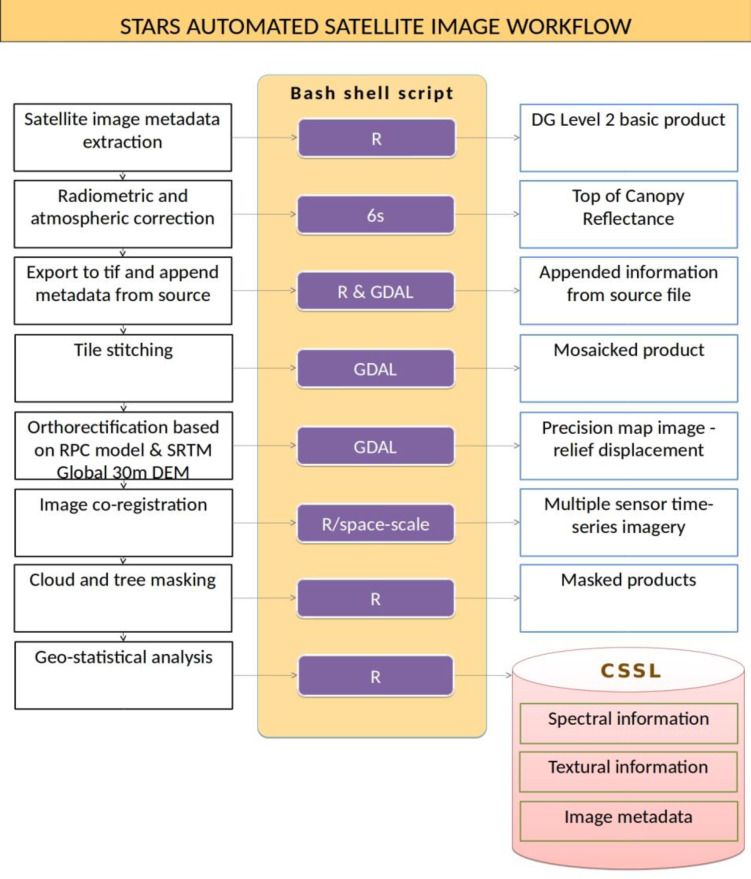
The processing chain of the STARS automated satellite image workflow.

The overarching idea is to create a fully automated system that processes the dataset and derives the required output. A single stream and single core process is followed for the first steps of image processing, where the earlier discussed pre—processing and dependencies are handled. Once the workflow of an image reaches a higher level product, the output is extracted depending on the scope of the task. The overall scheme is provided in Figure 3. The process starts with atmospheric correction using the 6S radiative transfer model. The produced .hdf file is converted to a GeoTIFF format, and the metadata from the original file are appended. The fourth step is a stitching operation of the tiles provided in a single delivery; for a DG delivery of 10 × 10 km^2^ area, this is often a number of four tiles. Subsequently, orthorectification using the NASA Shuttle Radar Topography Mission (SRTM) Version 3.0 Global 1 arcsecond DEM is implemented in GDAL based on the .rpc coefficient file (named .rpb in DG deliveries). Next, image registration based on a master image for each site is implemented to ensure a high coregistration accuracy. The last two steps of our image processing are the derivation of cloud— and tree masks. This is the highest level of post—processed image, based on which further scientific analysis can be carried out on calibrated data. Moving a step forward, timestamped statistical moments of the spectra and textural features are extracted for each target object such as a farm field, which are then collected in a crop spectral library.

Since studies of smallholder farming are expected to also be taken up in low—income countries, we wanted to make it possible for local research and operational organizations to process satellite data with free access and minimal costs. Commercial software has certain disadvantages such as licensing restrictions for end—users, an inability to freely access the source code, long—term maintenance, and security and transparency [29]. All these issues are more easily dealt with in free and open—source software.

**Table 2:** The satellite products processed and the associated image size and time required to run the individual steps of the workflow.

Image Sample	Satellite Sensor	Number of Bands	Ground Sampling Distance (m)	Number of Pixels	Delivery Size (MB)	Processing Time (min)	Atmospheric Correction	Subset and Mosaic	Tie Point Detection	Image Matching	Tree Mask	Spectral Statistics	Textural Statistics 64 gl	Textural Statistics 256 gl
1	WV—2/3	8	2	16,777,216	400	60	42%	2%	51%	2%	2%	0%	—
1b	WV—2/3 pan	1 (pan)	0.5	2,016,020,167	750	140	—	1%	—	0%	6%	—	27%	65%
2	RE	5	6.5	116,967,735	960	30	0%	8%	62%	5%	21%	0%	—	3
QB	4	2.4	41,933,143	106	40	54%	2%	37%	2%	2%	0%	—
3b	QB pan	1 (pan)	0.6	210,857,584	405	60	—	0%	—	1%	14%	—	22%	59%
4	GeoEye	4	2	50,405,041	200	52	36%	2%	58%	1%	2%	0%	—
4b	GeoEye pan	1 (pan)	0.5	406,566,720	780	68	—	1%	—	0%	5%	—	23%	71%

The workflow was initially developed for the DG data and was later adapted for RapidEye images without serious impediments. The decision to first address DG data was inspired by the finer spatial resolution of these images and in anticipation of more fundamental challenges. For instance, [30] developed an automatic geometric processing workflow for VHSR images based on roads with the same data sources and started with the development of software that could handle RapidEye data first, which subsequently raised problems in terms of adapting this to finer resolution data. The output type of the processing steps, in cases in which these are images, is GeoTIFF in order to accommodate the ease of ingestion and the processing of the intermediate products from end—users.

### 2.3 Module Description

In this section, we describe in detail the workflow steps. For a successful run of the workflow, the following datasets must already be available to the system:

1. High spatial resolution satellite images of the study area. In cases in which we have an image time series, one of the images is declared as the master image, onto which all other images are geometrically registered.
2. A DEM that covers the satellite image footprint and that is used in the orthorectification phase, if that phase is necessary.

If these datasets exist, the sequential processing steps described below, controlled through a bash script, can be run successively. An alternative approach that makes use of the same scripts is provided with make, and we discuss this in Section 3.4.

#### 2.3.1 Atmospheric Correction

Satellite sensors record the extra—terrestrial radiation flowing into the sensor, a measurement that is not directly associated with a physical quantity of the study material as it depends on the atmospheric conditions at the time of image acquisition and the sensor—target illumination geometry. While, for certain applications (e.g., land cover classification), this is less important, when comparing pixel spectra between images acquired on different dates/sensors or with ground measurements, it is crucial to work with consistent, calibrated products such as surface reflectance [31].

Several algorithms have been developed in the past to compensate for atmospheric effects such as the 6S model—based algorithm developed by NASA [32,33], the Sen2Cor algorithm [34] developed by the German Aerospace Center (DLR), and the MACCS (Multisensor Atmospheric Correction and Cloud Screening) algorithm developed by the French Space Agency (CNES) [35]. In our workflow implementation, we embedded the 6S model and revised and adapted it to accommodate the needs of the available image collection. The aerosol inversion relies on the assumed relationship between the surface reflectance in the blue, red, and near—infrared bands, using an algorithm similar to the one used for the MODIS aerosol product. It is described in more detail in [33]. The code is run natively by a FORTRAN program, and, for workflow embedding purposes, a Python wrapper was built around it. This module automatically downloads the auxiliary datasets required for its execution (water vapor, ozone, coarse resolution DEM obtained from the MODIS processing archive). It integrates the datasets with the selected types of satellite image to produce a common set of outputs. Our atmospheric correction module produces two outputs: the top—of—atmosphere and the surface reflectance. The latter is used in further processing. Occasionally, artifacts over water pixels are present in landscapes with irrigated agricultural areas, as the aerosol retrieval method is only valid over land pixels.

#### 2.3.2 Tile Stitching

DG imagery is delivered in bundles, each containing adjacent tiles. To form a single image covering the area of interest, the tiles are stitched together at this second step. Both R (‘mosaic’ function of ‘raster’ package) and GDAL (gdalwarp function) have algorithmic implementations that are required to perform this step. The results obtained from these algorithms are identical, and this also holds for the proprietary software results (i.e., ERDAS Imagine), with which they have been compared. In the current implementation, GDAL is used. RapidEye scenes are not undergoing this step as the images, either in the 2A or 3 processing level, are delivered as a single image covering the whole scene.

#### 2.3.3 Orthorectification

Orthorectification compensates for sensor orientation and terrain displacement through a geometric transformation onto a projected coordinate system. The required input is the satellite image, its corresponding RPC files, and a DEM that covers the satellite scene.

Satellite image products are typically delivered in the Standard2A or ORStandard2A processing level, which means that the pixel coordinates are not translated into geographical positions. Some deliveries took place in 3A format, so these were already orthorectified. Off—nadir angles range considerably, and are as high as 30 degrees in record images in side—looking configurations in areas of high cloud density; it is an important cause of inconsistent georeferencing between images. One is generally advised to work with off—nadir angles below 20 degrees [36], otherwise the images will present problems in plant canopy studies. Our orthorectification is applied to all images that are not yet orthorectified.

Several solutions exist for performing this task, and, in a study [37] that compared orthorectification methods for optical satellite data, it was found that ENVI, Erdas Imagine, PCI Geomatics, and Xdibias provided very similar absolute geometric accuracies; in our workflow implementation, GDAL is used. The DEM is used to reduce localization errors induced by jagged surface topography. A visual investigation of the Advanced Land Observing Satellite (ALOS) DEM provided by JAXA and the SRTM DEM by NASA, both freely available and with resolution of 1 arc—second (30 m), revealed that these two datasets are similar in the STARS use case areas. Eventually, we selected the SRTM because the ALOS DEM is, in essence, a Digital Surface Model (meaning that it does not account for understory vegetation height), and it is filled—in from other DEMs in areas with cloud coverage. The needed SRTM tiles covering our use case areas were downloaded in GeoTIFF format from the EarthExplorer website, stitched together, subsetted to the areas of interest, and projected onto a map coordinate system (WGS 84/respective UTM zone).

We evaluated the geometric accuracy of a delivered WV—3 ORStandard2A image acquired on 18 May 2015 at 10:46 over Sukumba, Mali, the respective orthorectified product, and the co—registered product on a master image, as described in Section 2.3.4. The comparison was made against 41 Ground Control Points (GCPs) recorded in the field with a differential GPS device (Table 3). We used the 30 cm panchromatic image for better clarity to distinguish the GCPs cross marks. The results present considerably lower RMSE_X_ and RMSE_Y_ for the co—registered image (1.547 m and 0.361 m respectively), in comparison to the raw image (6.616 m and 1.886 m respectively). However, when the latter was orthorectified, it produced a similar RMSE_Y_ (1.521 m) but a higher RMSE_X_ (11.527 m). Looking into the mean and standard deviation of the absolute residual values, it is clear that the orthorectified product has a higher mean but a considerably lower standard deviation of the absolute residual value, which translates into a larger average misregistration but, at the same time, a consistent shift throughout the image, contrary to the raw product, which has higher standard deviations and residuals across the images that are variable. Obviously, the co—registered (i.e., to a master) image produces an overall RMSE that is lower than the spatial resolution of the specific multispectral image, which is 1.2 m.

**Table 3:** A statistical comparison of the three image products against 41 Ground Control Points (GCPs).

Statistical Attribute	Image Provider	Orthorectified	Co—Registered
Mean_X_ of absolute residual values (m)	5.288	11.375	1.319
Standard deviation_X_ of absolute residual values (m)	4.026	1.888	0.82
Mean_Y_ of absolute residual values (m)	1.875	1.511	0.324
Standard deviation_Y_ of absolute residual values (m)	0.205	0.169	0.162
Min_X_ residual (m)	−0.136	−1.084	0.046
Max_X_ residual (m)	17.1	−13.527	2.872
Min_Y_ residual (m)	1.416	1.086	−0.004
Max_Y_ residual (m)	2.318	1.785	0.635
RMSE_X_ (m)	6.616	11.527	1.547
RMSE_Y_ (m)	1.886	1.521	0.361

#### 2.3.4 Image Co—Registration

Although orthorectification compensates for geometric distortions due to relief and imaging geometry, if done without GCPs, as described above, the process provides an absolute accuracy that may not suffice for certain applications. Mapping crops in smallholder fields with VHSR images is such an application, as the risk of spatial misalignment between images is high. Significant accuracy of georeferencing can be achieved by bias correction of the RPC parameters prior to orthorectification to eliminate systematic effects [38]. However, this requires the selection of GCPs per image, which is unsuitable for automated applications. Several image matching algorithms have been established to automatically detect candidate GCP points, used mainly in pattern recognition, such as in the Scale—Invariant Feature Transform (SIFT) algorithm introduced in [39,40] and the Speeded Up Robust Features (SURF) of [41].

An application of these methods to VHSR images provided shifts greater than the pixel size of the image and were disregarded. Instead, an algorithm that implements feature extraction with automatic scale detection [42] based on the identification of objects was developed in—house. The overall idea is based on GCP extraction from an image time series, which is a well—known problem due to the temporal variability of the position of the candidate objects [43]. Roads (excluding bridges and elevated roads) may provide reliable GCPs, and these have been used as GCPs in several studies [12,44]. However, low—income countries typically feature a sparse paved road network, and concrete roads are regularly absent from the image scene. In our approach, tree crowns are characterized by a bell—shaped intensity surface [45] and corrected for parallax. Their centroids are extracted as tie points, and the strongest candidates are used as GCPs. For every study area, one image is declared as the master image on the basis of the longest visible shadow of the trees, as calculated from the positions of the sun and the satellite from the image metadata [46].

The level of georeferencing accuracy does not only associate with the image’s spatial resolution but also with the intended application. Generally, however, a high accuracy is important in the analysis of large data volumes because of easier integration with Geographical Information System applications and post—processing [29]. In their algorithmic development of the automated extraction of GCPs from the road network [12], the authors report a higher accuracy of coregistration of RapidEye images in comparison to DG images. They attribute this to the improvements of their initial algorithm for RapidEye, which extracts the road centerline from an image with a pixel resolution of one to three pixels of the width of the road. As the spatial resolution increases, the number of pixels required to cover the road width also increases, and, therefore, the centerline is no longer pronounced; instead finer road features become discernible. This methodology suggests that GCPs derived from the road network are dependent on the correspondence of the spatial resolution of the road width and the pixel resolution. In our approach, however, the higher the spatial resolution, the higher the accuracy.

#### 2.3.5 Cloud Masking

Clouds and haze are impediments when working with optical satellite data, especially in cloud—prone areas such as the tropics (for instance, a quantitative analysis of cloud cover on Landsat data over Brazilian Amazonia from 1984 to 1997 is given in [47]). In land cover applications, cloudy and heavily hazy areas are normally excluded from further processing as they considerably contaminate the spectral signature of areas of interest.

Traditionally, cloud masking makes use of a thermal band. However, VHSR sensors do not come with such, and, when available in medium resolution it is of a coarser resolution than the other channels (e.g., the spatial resolution of Landsat 7 ETM+ is 60 m while that of Landsat 8 TIRS is 100 m [48]). Some alternatives proposed to address this limitation have been the use of Markov random fields [49], linear spectral unmixing [50], pixel—based seed identification and object—based region growing [51], and a multi—temporal approach at constant viewing angles [35]. Some cloud—specific masking algorithms are the AFAR algorithm (ACOLITE/FMASK Aquatic Refined) developed by the Royal Belgian Institute of Natural Sciences (RBINS) , the Automatic Cloud Cover Assessment modified (ACCAm) algorithm (ACCA modified) by VITO, and Idepix developed by Brockmann Consult GmbH.

We tested several algorithms for cloud detection in the different landscape environments and concluded that they largely depend on the radiometry of image and background (i.e., the land cover radiance). It is therefore difficult to identify a single best method for automation without fine—tuning that will provide a universally acceptable cloud mask for any arbitrary study area. None of the discussed methods yielded accurate results in all cases. Our implementation assumes that pixels corresponding to clouds have the highest reflectance in short wavelength bands within the scene; this hypothesis is on par with other thresholding techniques for cloud masking. It is a universal approach that is suitable for the purpose of study and the areas of interest as smallholder farming prevails in relatively low latitudes; however, in cases of snowy, icy, or other high reflective areas in the landscape, the cloudy pixels will not be the brightest pixels, which renders this technique unsuitable. Under this assumption, the problem of detecting cloudy pixels is reduced to locating the brightest pixels of a shortwave band and deriving the percentage of cloud cover within the scene. The threshold of the percentage of cloud pixels is set by the metadata of each image, which lists the percentage of pixels corresponding to cloud for each delivery. In regard to the selection of the appropriate shortwave band, a comparison between the blue and cyan channels for detecting the brightest pixel values led to no substantial difference, and the blue channel was eventually chosen. The cloud mask, therefore, contains the pixels with the highest reflectance value in the blue band determined by the histogram and that fall within the percentage of cloud coverage reported in the metadata of the image delivery.

#### 2.3.6 Tree Masking

At the spatial resolution at which we are investigating images, trees appear as individual objects and can thus be identified and spatially isolated. This allows us to eliminate their spectral contribution from that of the farm fields and to determine pure soil or crop spectra. The tree mask is taken from the image coregistration step, as described in Section 2.3.4. We apply a lowered NDVI threshold to eliminate false negatives. A tree mask based on the blobs detected is built on a master image and then adapted for other images, using the image’s nadir angle to account for sensor—target geometry.

Our method provides a detailed tree crown in the case of DG data. However, for RapidEye data, due to coarser resolution, the degree of tree crown detection is lower. This sets a case for using a single high resolution image in the season combined with a time series of lower resolution images. Our method works well for isolated tree crowns but not sufficiently well for tree rows or (small) forest patches. This presented problems in the Bangladesh study area, where isolated trees are scarce and trees commonly align the field edges.

## 3 Results and Discussion

**Figure 4:**
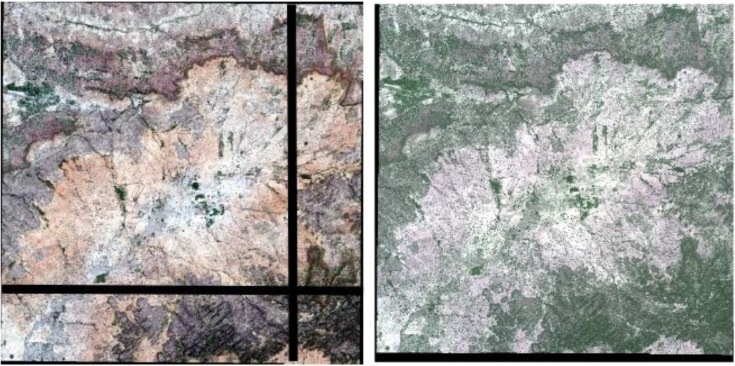
Raw data (**left**) and a stitched, radiometrically, and atmospherically corrected, orthorectified, co—registered, and tree/cloud masked image (**right**).

Our workflow provides, in automatic fashion, radiometrically and atmospherically corrected, stitched, orthorectified, and coregistered images with associated cloud and tree masks (Figure 4). This is the beginning of further parallel processing routines that yield numerous qualitative and quantitative results and offers use as value—added product in applications such as precision agriculture (e.g., [4]). Here, we present three post—processing branches for utilizing the application—ready dataset of the basic workflow. First, we describe an application of the image workflow that extracts spectral and textural statistics from individual fields and aggregations of fields. The second application demonstrates the information available from the workflow and data feeding a crop spectrotemporal library. Last, the near—real time phenological development of a crop is presented through the use of a time—series.

### 3.1 Field and Crop Statistics

One important use of the image workflow is the extraction of spectral and textural statistics for the characterization of crops and fields. This operation requires satellite images that are radiometrically and atmospherically corrected and that are highly aligned over time and over the fields. Otherwise, the extracted statistics from such small fields will be incoherent. For spectral statistics, we use multispectral images, and, for textural statistics, we use panchromatic images.

To obtain pure results per field, a (negative) buffer of 2 m is applied to disregard potentially fuzzy pixels at the field boundary; this eliminates vegetation along the edges of the field to a large extent. Cloud and tree masks are applied at this stage. The first four statistical moments (i.e., pixel count, mean, variance, and skewness) of the surface reflectance contained within the field polygon are extracted, as well as the correlation and covariance matrices between the multispectral bands. Table 4 lists the spectral attributes extracted per field and per satellite image, and their values are delivered as a single .csv file for each image. Concurrently, the mean values and standard deviations of nine popular spectral vegetation indices are computed (NDVI, green NDVI, EVI, TCARI, NIR red, SARVI, SAVI, MSAVI, WV2) (Table 5).

**Table 4:** Spectral statistical moments extracted from the images.

Statistical Moment	Explanation
0	Number of pixels within the polygon
0	Number of pixels contributing to the statistics (masked pixels excluded)
1	Mean
2	Variance
3	Skewness
mixed	Band—to—band correlation (i,j)
mixed	Band—to—band covariance (i,j)

**Table 5:** Empirical indices proposed frequently in remote sensing of agriculture.

Index	Reference	Formula
NDVI (Normalized Difference Vegetation Index)	[52]	$\frac{R_{NIR}-R_{R}}{R_{NIR}+R_{R}}$
Green NDVI (Green Normalized Difference Vegetation Green Index)	[53]	$\frac{R_{NIR}-R_{G}}{R_{NIR}+R_{G}}$
EVI (Enhanced Vegetation Index)	[54]	$\frac{2.5*R_{NIR}-R_{R}}{R_{NIR}+6*R_{R}-7.5*R_{B}+1}$
TCARI (Transformed Chlorophyll Absorption Ratio Index)	[55]	$3\left[\left(R_{\text{REDGE}}-R_{R}\right)-0.2\left(R_{\text{REDGE}}-R_{G}\right)\left(\frac{R_{\text{REDGE}}}{R_{R}}\right)\right]$
Simple ratio (NIR/RED)	[56]	$\frac{R_{NIR}}{R_{R}}$
SARVI (Soil and Atmospherically Resistant Vegetation Index)	[57]	$\underset{\text{where }R_{RB}=R_{R}-\text{gamma }\left(R_{B}-R_{R}\right)}{\frac{\left(1+L\right)\left(R_{NIR}-R_{RB}\right)}{\left(R_{NIR}+R_{RB}+L\right)}}$
SAVI (Soil Adjusted Vegetation Index)	[58]	$\frac{\left(1+L\right)\left(R_{NIR}-R_{R}\right)}{\left(R_{NIR}+R_{R}+L\right)}$
MSAVI2 (Improved Soil Adjusted Vegetation Index)	[59]	$\frac{2R_{NIR}+1-\sqrt{{\left(2R_{NIR}+1\right)}^{2}-8\left(R_{NIR}-R_{R}\right)}}{2}$
NDVI (Normalized Difference Vegetation Index) based on NIR2	[52]	$\frac{R_{NIR2}-R_{R}}{R_{NIR2}+R_{R}}$

Textural attributes are derived from the panchromatic images using the Gray—Level Co—Occurrence Matrix (GLCM) [60], which is a statistical approach to texture characterization suitable for regions with irregular shapes [61]. This applies well to smallholder fields. GLCM was calculated for four angles to cater for different directions along and perpendicular to the sowing rows. The following thirteen texture descriptors are derived: angular second moment, contrast, correlation, variance, local homogeneity, sum average, sum variance, sum entropy, entropy, difference variance, difference entropy, information measures of correlation, and maximal correlation coefficient. All of these were determined with 64 and 256 grey levels. The texture statistics are delivered in a separate .csv file for each image and for both of the two grey level classes.

### 3.2 Crop Spectrotemporal Signature Library (CSSL)

The wealth of information derived from a large satellite image collection can be efficiently utilized only if the data are stored in a platform with a clear and organized structure, where users can query and access the data needed. This objective is served by the Crop Spectrotemporal Signature Library (CSSL), which is a database containing the spectral and textural information described in the previous section, accompanied by metadata and in—situ observations whenever available. In the framework of the project, 4.5 TB of VHSR satellite data have been acquired and archived; while this is, by and large, proprietary data, the information extracted out of it such as the field spectra is not. Hence the CSSL is acting as an open—access information platform, where a user can query, explore, and acquire a time—series of crop—specific VHSR spectra.

With the CSSL, we aim to realize a publicly available information resource that supports others in image—based research on smallholder agriculture. Eventually, all data and all code around it will be available to research teams with such stated interests, and our team will support its use and further expansion. Our ambition is to make it a valuable public global good that serves to address, study, and understand the intrinsic heterogeneity of smallholder farming.

### 3.3 Monitoring Crop Phenology

Another application is vegetation dynamics, which allows us to monitor the seasonal progression of vegetation. Coarse resolution satellite images have been used extensively to study land surface phenology at continental to global scales (e.g., [62]). However, smaller ecosystems are particularly important as they are associated with their environment and hence can be more easily interpreted and associated with the local ecosystem.

**Figure 5:**
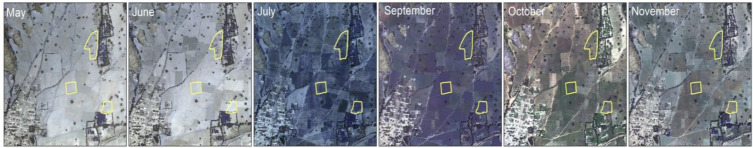
The temporal evolution of individual farms as depicted from true—colour composite VHSR satellite data within the growing season in Sukumba, Mali.

**Figure 6:**
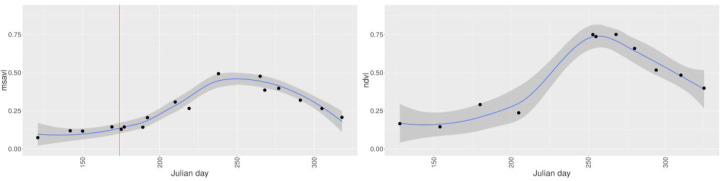
Temporal progression of vegetation indices (average (blue line) and standard deviation (shaded)) for a single crop field based on all the VHSR satellite images (from WV—2, WV—3, QuickBird, and GeoEye) for a whole year processed in the workflow. MSAVI for a cotton field in Sukumba, Mali for the year 2014, with a red vertical line indicating the sowing date (**left**), and NDVI for a soybean field in Kofa, Nigeria, for the year 2015 (**right**).

**Figure 7:**
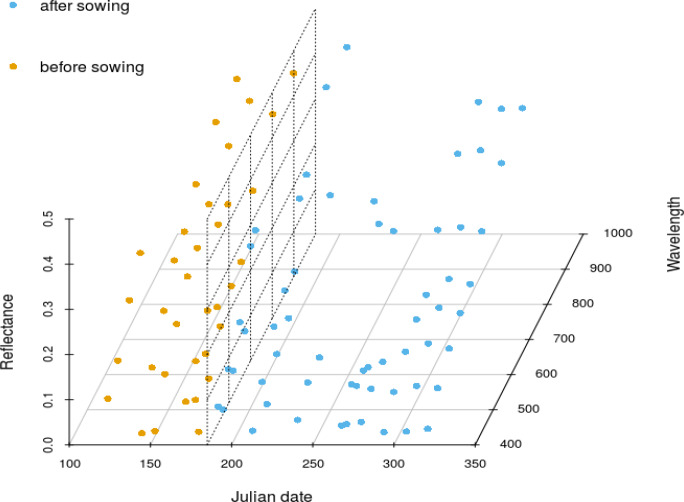
Temporal evolution of the eight—band spectral signature of a single crop. The spectral signatures for the acquisitions before and after sowing are depicted in different colors. The dotted plane parallel to yz represents the concurrent ploughing and sowing date for this field (185 Julian day of the year 2014).

**Figure 8:**
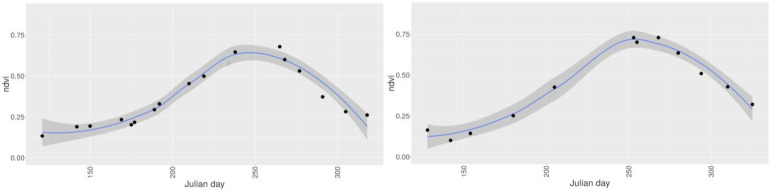
Temporal progression of NDVI (average (blue line) and standard deviation (shaded)) for all the fields for a specific crop (cumulative statistics) based on all the VHSR satellite images processed in the workflow. Statistics for all peanut fields in Sukumba, Mali, for the year 2014 (**left**) and for all maize fields in Kofa, Nigeria, for the year 2015 (**right**).

In Figures 5–8 we demonstrate the provision of rapid and precise (in terms of radiometry and spatial distribution) information on land surface phenology for smallholder farm fields. More precisely, Figure 5 shows the evolution of individual fields within the crop season as captured by DG satellites, while Figure 6 presents the progression of vegetation indices for two fields in Mali and Nigeria for the years 2014 and 2015, respectively. Figure 7 depicts a 3D spectro—temporal spectral signature representation indicating the eight—band signatures for the acquisitions for two classes of interest, before and after the sowing date for a single farm. Based on these field—specific metrics, regional statistics can be derived. For instance, in Figure 8, we show the average and standard deviation of NDVI from all available images processed from the workflow for all peanut fields in Sukumba, Mali, for 2014 and for all maize fields in Kofa, Nigeria, for 2015. In this figure, users can observe the phenology of a specific crop for a given area cumulatively and derive information on crop statistics for a larger area instead of an individual farm. A total of 17 and 12 observations were collected for the Mali and Nigeria fields, respectively. This indicates that, in spite of using all the available DG satellites, the number of cloud—free images acquired from this constellation in the typical climatic conditions of the tropical savanna is frequently constrained by cloud coverage. The results indicate a fair agreement with the spectral evolution of the crop cycle and could possibly be improved further by a cross—sensor calibration inclusion in the workflow, which is planned for the future. While the image footprints of DG and RapidEye data are not large enough to cover a whole country, data from Sentinel—2 could potentially be ingested in this workflow to address the need for countrywide information. However, the limitations imposed by the coarser spatial resolution have to be examined and addressed; for instance; does the tree detection work at a ground sampling distance of 10 m, and can smallholder farms be confidently quantified by a representation of a smaller number of pixels?

### 3.4 Alternative Workflow Implementation through GNU Makefile

The original implementation of the STARS image workflow is a Linux shell script. At an early stage in the design and realization of our workflow, we understood that our approach had to be modular and that the resulting image products might come in versions with the need for reprocessing when new routines became available or when ancillary input data were updated. As the computational cost for complete reprocessing cycles is prohibitive, we needed an approach that would allow the rerun of only the necessary processing steps. Specifically, we wanted to routinely allow the:

- addition of image sources to the collection and the automatic processing of only these by the workflow,
- the replacement of a workflow step and have this lead to reprocessing of image half—products leading to up—to—date end—products, and
- the parallelization of the execution of the workflow through intelligent rules.

We achieved the above using a GNU Makefile, which is a facility to define file—oriented input/output processes and associated file dependencies through rule—based specifications [63]. Originally devised to help in software development projects, it serves well to define more general computer processes like our image workflow. An example Make rule depicting the implementation of a processing step is shown in Equations (1) to (3).

# Determine the files that will be atmospherically corrected. The output of the “find” call is placed in the list.

1 atcor_list_src := $(shell find ~/stars/acquired/DG/ -name '*.TIF' | sort)

# Generate the corresponding full paths of the output files. Copy the 'atcor_list_src', but substitute 'acquired/DG' by 'derived/1_atcor_6s'.

2 atcor_list_dst := $(join $(subst acquired/DG,derived/1_atcor_6s,$(abspath $(addsuffix../,$(atcor_list_src))))),$(addprefix /,$(notdir $(atcor_list_src))))

# Atmospheric correction: add one Makefile rule per src/dst file pair. “make” will determine if the 'dst' file needs to be (re—)created, and the recipe is a call to pythonWrV8.py

3 $(call addrule,$(atcor_list_src),$(atcor_list_dst),cd $(base)/atcor_6s/PythonWrapper &&python $(base)/atcor_6s/PythonWrapper/pythonWrV8.py –band4n8alldir=$$(dir $$<)–outputdir=$$(dir $$@))

Specific challenges had to be resolved for our implementation with Make. Image providers use non—trivial and non—standardized filename conventions. Make was originally meant to operate in a flat folder structure, with the file name prefix, stem, and extension as primary keys to the file’s semantics. Source and target files can be referred to by a filename pattern, but this mechanism allows only one wildcard. Tile stitching, as a many—to—one image process, is specifically cumbersome because many tiles should produce a single image mosaic, but the individual tile filenames do not make explicit the spatial relations between tiles that one needs to understand for stitching. In other words, Make is not spatially aware. Our solution to these challenges is to devise the file dependency rules on the fly: one Make statement constructs the actually required Make—rules, as opposed to a single rule with wildcards. These rules are subsequently executed. We add spatial intelligence to this process by calling R scripts, capable of reading and processing image metadata, directly from the image or by reading the scene metadata from the CSSL database, which provides the needed file—dependency information to the “Make” utility.

## 4 Conclusions

An automated satellite image processing workflow for smallholder agriculture based on free software has been developed and is presented here. The key characteristics are the automated capability, the delivery of near—real—time application—ready products, the free and open—source nature of the algorithmic approach, and the development tailored to smallholder agriculture. This workflow can be set up on a Linux machine and requires no cost for direct or indirect software licensing. The individual steps have been presented, and the adjustments made to accommodate smallholder—specific analysis have been discussed. It supports VHSR satellite images and is currently being tested to see if it can accommodate the use of Sentinel—2 images. Calibrated data and efficient and timely processing of the vault of satellite images that are gradually becoming available is important for shifting from a data—centric to a product—centric solution based on remotely sensed images. This work has demonstrated how an automated workflow, once set up, can deliver, in near—real—time, information to end—users and decision makers, who need no remote sensing knowledge to apprehend the results. However, despite the wealth of information produced, the value of the product can be appreciated only if the information aids a decision resulting in higher profitability [4].

In order to adequately monitor the temporal evolution of smallholder farms with remote sensing, a high spatial and temporal resolution dataset is needed. This requires new advancement in satellite remote sensing; an attempt to integrate free data satisfying the requirements for smallholder agriculture (e.g., Sentinel—2, Venμs and SPOT—5) can enrich the available information vault. An investigation of the usability of Unmanned Aerial Vehicles (UAV), a development signaling a new era in remote sensing regarding spatial accuracy, temporal frequency, and radiometric quality, and similar automated workflows encompassing virtual constellations could be a sound basis, not only for delivering near—real—time information, but also for delivering results for further scientific analysis.

The Faculty of Geo—Information Science and Earth Observation (ITC) team aims to publish the presented workflow as an open—source, global public good, together with its partner crop spectro—temporal signature library (CSSL). We invite research and development teams with an interest in the image—based monitoring of smallholder farming to collaborate with us and to make use of this workflow for their work. Teams that have an interest in such collaboration should write to Rolf A. de By at r.a.deby@utwente.nl.
